# What motivates open defecation? A qualitative study from a rural setting in Nepal

**DOI:** 10.1371/journal.pone.0219246

**Published:** 2019-07-01

**Authors:** Navin Bhatt, Shyam Sundar Budhathoki, Don Eliseo III Lucero-Prisno, Gambhir Shrestha, Meika Bhattachan, Jeevan Thapa, Avinash K. Sunny, Pawan Upadhyaya, Anup Ghimire, Paras K. Pokharel

**Affiliations:** 1 B. P. Koirala Institute of Health Sciences, Dharan, Nepal; 2 London School of Hygiene and Tropical Medicine, London, United Kingdom; 3 University of the Philippines Open University, Laguna, Philippines; Tribhuvan University Institute of Medicine, NEPAL

## Abstract

**Introduction:**

Open defecation is ongoing in Nepal despite the rise in efforts for increasing latrine coverage and its use. Understanding the reasons for open defecation would complement the ongoing efforts to achieve the ‘open defecation free’ status in Nepal. This study aimed at exploring different motivations of people who practice open defecation in a village in Nepal.

**Methods:**

This study was conducted among the people from the Hattimudha village in Morang district of eastern Nepal, who practiced open defecation. Maximum variation sampling method was used to recruit participants for 20 in-depth interviews and 2 focus group discussions. We adopted a content analysis approach to analyze the data.

**Results:**

We categorized different reasons for open defecation as motivation by choice and motivation by compulsion. Open defecation by choice as is expressed as a medium for socializing, a habit and an enjoyable outdoor activity that complies with spiritual and religious norms. Open defecation by compulsion include reasons such as not having a latrine at home or having an alternative use for the latrine structures. Despite having a private latrine at home or access to a public latrine, people were compelled to practice open defecation due to constraints of norms restricting latrine use and hygiene issues in general. For women the issues with privacy and issues refraining women to use the same latrine as men compelled women to look for open defecation places.

**Conclusion:**

Open defecation is either a voluntary choice or a compulsion. This choice is closely linked with personal preferences, cultural and traditional norms with special concerns for privacy for women and girls in different communities. The ongoing campaigns to promote latrine construction and its use needs to carefully consider these factors in order to reduce the open defecation practices and increase the use of sanitary latrines.

## Introduction

Every year 525,000 children die due to diarrhea out of the 1.7 billion cases of childhood diarrhea worldwide [1]. Poor sanitation is responsible for 10% of the global disease burden, causing diarrheal diseases, neglected tropical diseases, acute respiratory diseases and malnutrition among children [2].

Basic sanitation facilities are accessible to only 68% of the global population [3]. Among 2.3 billion people who lack access to toilets or latrines, 892 million still defecate in the open places [3]. Higher under-five mortality and malnutrition rates are reported in countries which have higher proportions of people practicing open field defecation [3]. Open defecation is also associated with violence against women in the low middle-income countries (LMIC) [4] including rape among women and girls [5].

The United Nations calls for eliminating open defecation by 2025 [6]. Open defecation has been incorporated in the Sustainable Development Goals (SDG) in target 6.2 aiming to “achieve access to adequate and equitable sanitation and hygiene for all and end open defecation, paying special attention to the needs of women and girls and those in vulnerable situations by 2030” [7]. There are different approaches to increase the construction of latrines and promote its use around the world. ‘Sanitation marketing’, involves interventions on raising awareness and motivating the households to adopt and use latrines [8]. Community-led total sanitation (CLTS) approach mobilizes the whole community to gain a social status of “open defecation–free” community [9,10]. ‘Community health clubs’ raises awareness through health debates in the communities [11], and ‘Sanitation as a business’ approach mobilizes the local private sector in latrine building and maintenance [12]. School Led Total Sanitation (SLTS) focuses on motivating the communities and students towards behavioral transformation and latrine promotion, building upon the enhanced partnership of school, local level organizations and the community [13].

Among the world’s population who practice open defecation, more than two-thirds (558 million) live in the rural areas of South Asia. With continuous efforts, the proportion of people practicing open defecation in South Asia dropped from 65% in 1990 to 34% in 2018 [14]. Despite the efforts through different approaches, attaining and sustaining the ‘open defecation free’ status has been possible only in less than half of the places globally [15]. The available quantitative data on open defecation provides limited information regarding the motivations for open defecation [14,15]. A qualitative study from Zambia suggests that the preference for defecation practice of either using a latrine or going for open defecation may have deep-rooted cultural influences [16].

### Current situation of sanitation in Nepal

Nepal is a low-income country in South Asia with a population of 28 million with 83% of the population residing in the rural areas and one-fourth of the population living below the poverty line [17]. As per the 2016 Nepal Demographic Health Survey (NDHS), the under-five mortality and infant mortality rates are 39 and 32 deaths per 1000 live births respectively [18] with diarrhea as the major cause of childhood morbidity and mortality in Nepal [19]. According to NDHS 2016, 62% of the households in Nepal have access to an improved sanitation facility, 22% people have access to shared facilities, 1.6% have access to unimproved facilities and about 15% have no facility thus use open space for defecation [18]. Improved sanitation facilities include any non-shared toilet of the following types: flush/pour flush toilets to piped sewer systems, septic tanks, and pit latrines; ventilated improved pit (VIP) latrines; pit latrines with slabs; and composting toilets. Unimproved facilities include shared toilets that are used by more than 2 households or flush/pour toilets that are not connected to piped sewers, septic tanks and open pit latrines [18]. The proportion of open defecation practice is higher in rural areas than in urban areas (21% vs. 11%). Open spaces for defecation include behind the bushes, open fields and the river banks [18]. Inadequate sanitation is a major factor responsible for diarrhea cases in children in Nepal [20].

Government and non-government agencies began efforts to increase latrine coverage in Nepal since 1990, followed by CLTS in 2003 and SLTS in 2006. The latter approaches emphasized on creating open defecation free (ODF) communities in Nepal. Nepal Government emphasizes that 'Open defecation free' area is an area with no feces exposed to air. Thereby an 'ODF' status is given to an area if there is no open defecation in the designated area at any given time; all households have access to improved sanitation facilities (toilets) with full use, operation, and maintenance; and all the schools, institutions or offices within the designated areas have toilet facilities [21]. Beginning with the first ODF village in 2007 with Kaski district as ODF district in 2011, Nepal government aimed to make Nepal ODF by 2017 by implementing the Sanitation and Hygiene master plan 2011 [22]. As of March 2018, based on the accessible latrines as built by the community through the government support, only 49 out of 77 districts are ‘open defecation free’ status given by the government [23]. The data reflects the population that has access to the latrines. However, there is insufficient data on the latrine utilization rates.

A report from India suggests that open defecation is reported even from areas with high latrine coverage [24] with another literature from India reporting almost 40% of the households with latrines having at least one member from their family practicing open defecation [25]. Therefore, it is questionable whether the districts declared ODF would be truly an open defecation free zone in Nepal. This is also reflected from a previous study in Nepal, which reports 5.7% of the households with latrines are still practicing open defecation [26]. Defecation practices are influenced by sanitary preferences of people [25], habits, cultural practices, availability of materials and resources [27], and different environmental and social stressors [28]. The available quantitative literature shows the latrine coverage, ODF prevalence and the factors affecting latrine utilization. However, little can be found on factors at a personal level that are associated with the ongoing open defecation practices. Understanding the social and cultural issues surrounding the practice of defecation using qualitative methods seem essential to support the ongoing sanitation campaigns and latrine promotion all over the country. Therefore, this study aimed to explore the reasons for practicing open defecation among the people living in a rural village from a low latrine coverage district in Nepal.

## Methods

### Ethics approval and consent to participate

Institutional Ethical Review Board of the B P Koirala Institute of Health Sciences (BPKIHS) approved this study as per the undergraduate research proposal approval process. Literate participants were given an information sheet that provided details on the research objectives, expected role of the respondents and the voluntary nature of participation. They were informed that their decision to participate or decline participation would not affect any benefits or services received by them. Written informed consent were obtained from all literate participants. For the ones who were unable to read, the information sheet was read out and the research objectives, expected roles of participants and voluntary nature of participation was explained in the presence of a literate elder community person. A thumbprint was obtained, and a witness countersigned this.

### The research team and reflexivity

The interview/facilitation team was comprised of experts from different fields with one medical student, two postgraduate resident doctors, and one public health academic researcher. An orientation session was conducted for the interviewers/facilitators using the final interview schedule and the topic guide. A one-week training on qualitative methods of interviewing, facilitating, note taking, recording and transcribing was conducted for the team.

The participants of the study belonged to Hattimudha village, which is part of the ‘community health service’ area of the institute (B. P. Koirala Institute of Health Sciences, hereafter BPKIHS). BPKIHS adopts a teaching district concept of community based medical education curriculum serving eight districts of eastern Nepal [29]. Hattimudha is in Morang district, which is about 30 kilometers form BPKIHS. The research participants did not know any of the researchers personally or professionally. However, the participants were aware that the researchers were from BPKIHS, which serves their community for health services. As hygiene and sanitation are linked with health, the residents are usually more comfortable talking about their experience with health professionals. The researchers had prior experience of the subject which came from a quantitative study on latrine utilization conducted earlier in this setting. The research reported latrine coverage of 75.9% with a utilization rate of 94.3%, which provided a basis for a qualitative exploration of the motivations for the open defecation among the people residing in Hattimudha [26].

### Study design

The study utilized qualitative design as opined by Miller and Crabtree who state that qualitative research has no prepackaged research design [30]. This design is justified for the current study, as the preferences of each individual for defecation is different due to individual choices and the prevailing cultural norms. We conducted this study from August 2016 to December 2017. The data was collected during the spring of 2017.

### Participant selection

We used a maximum variation sampling method to recruit the participants who practiced open defecation into the study. With preliminary characteristics of gender and presence of latrine at home, a brainstorming was conducted with community volunteers to generate a list of people who practiced open defecation [31]. Public announcement for recruitment was not suggested appropriate by the volunteers. The list was further added with more volunteers upon through recommendation of people by the initial respondents. Based on the list, the respondents were recruited taking into consideration other characteristics in Table 1. We recruited participants until we reached a point where the participant response did not yield any new information. This resulted in a total of 20 respondents, who participated in in-depth interviews (IDI) and 15 respondents participating in two focus group discussions (FGD). All the potential participants were approached including all who agreed to participate in this study.

**Table 1:** Socio-demographic characteristics of the respondents.

Characteristics		IDI (n = 20)	FGD (n = 15)
**Gender**	Male	13	8
	Female	7	7
**Education**	No School education	6	7
	Primary School	7	4
	Secondary and above	7	3
**Religion**	Hindu	12	9
	Others	8	6
**Family size**	≤5	11	7
	>5	9	8
**Economic status**	Below poverty line	8	9
	Above poverty line	12	6
**Presence of a child under-five at home**	Present	4	3
	Absent	16	12
**Latrine at home**	Present	6	4
	Absent	14	11
**Head of the household**	Yes	3	4
	No	17	11

### Study setting

The district of Morang has a latrine coverage of 87% [32]. As per a study [26] in 2017, the latrine coverage of Hattimudha is 75.9% with open defecation being practiced by 28.4% (24.1% who did not possess latrine at households and 4.3% of those who possessed latrine). Data was collected in a community setting either at the household or at a place as preferred by the research participants. To maintain the privacy of the participants and confidentiality of the information, it was ensured that no one other than the selected participants and the moderators attended the sessions.

### Data collection

A previous study conducted in the area along with the literature review was the basis for the preparation of the topic guide for FGD and the interview schedule. The research team finalized the topics in a workshop followed by an orientation to the data collectors before research implementation. Focus group discussions were used in addition to the in-depth interviews, which enabled triangulation of the data in this study to ensure the validity and reliability of the study. The IDI and FGD were guided by an interview schedule and topic guides consisting of open questions. The topics included preferences for defecation and reasons for making those choices for defecation. The participants were given choices to participate either in individual interview sessions or in the discussion as focus groups. We conducted the IDIs with respondents who reported open field defecation considering maximum variation sampling. We conducted the FGDs in two convenient groups of male and female participants based on their availability and comfort zone consisting of eight male members and seven female members.

There were two moderators moderating each of the focus group discussions. We trained the moderators who were familiar with the local language, to conduct, observe and record the FGDs. The study objectives and FGD moderation skills were briefed to the moderators through a one-day course. One moderator facilitated the discussion, while the other concentrated on note-taking and audio recording. The moderators switched roles for each discussion. The sessions were conducted in Nepali language as preferred by the participants. All interviews and FGDs were audio-recorded after seeking the consent of the participants. The interviews lasted between 25–40 minutes and the FGDs lasted for 50–80 minutes. All the FGDs took place at the Hattimudha Village Health post because of its central geographical location and availability of adequate space for the FGDs.

### Data analysis

The descriptive analysis of the socio-demographic characteristics of the respondents was done as illustrated in Table 1. A framework method [33] of content analysis and systematically organized the research data was used. The steps included transcription, familiarization with the interview, coding, developing a working analytical framework, applying the analytical framework, charting the data into the framework matrix and interpretation of the data. All four interviewers were involved in the transcription of the recorded audio. The session transcripts were translated into English before performing analysis line by line. Preliminary codes were assigned to the available data and using the codes common themes were searched. The research team finalized the themes after review of the preliminary themes forming the analytical framework. During the reporting of findings, the exact translation of the quotes from the participants was used. To ensure the consistency of the data and the findings, two authors were involved in data analysis and reporting. The quoted verbatim was accompanied by the interpretation of the findings in sentences. The interpretation and analysis of the data were completed in a closed group workshop in presence of all members of the team, where the excel sheet containing the coded data was presented on screen and input was received from all the members. The working analytical framework was then finalized and presented as a summary of the motivators for open defecation as presented in Table 2.

**Table 2:** Summary of the motivators for open defecation.

Motivations for open defecation		
By Choice	By Compulsion (Issues regarding Private Latrines)	By Compulsion (Issues regarding Public Latrines)
Socialization	Absence of latrine at home	Issues with queuing
Independent outdoor activity	Alternate use of latrine	Privacy issues for females
Habit	Hygiene and maintenance issues	Hygiene issues
Convenient choice	Household norms for latrine use	Cultural norms for latrine sharing
Religious beliefs	Cultural norms for latrine sharing	
Hygiene issues		

## Results

The respondents of the interviews and the focus group discussions were represented based on gender, education, religion, family size, economic status, having a child under-five at home, ownership of a latrine and status as a head of the household. The characteristics of the respondents can be found in Table 1.

The responses from the IDIs and FGDs were grouped into the three themes as motivations for open defecation by choice, motivations for open defecation by compulsion (issues regarding private latrines) and motivations for open defecation by compulsion (issues regarding public latrines). The motivations for open defecation are summarised in Table 2.

### Motivations for open defecation by choice

#### Socialization

Participants expressed defecation in open fields as an activity undertaken with friends, a common practice by people in the neighborhood that allows socialization.

"*I have been going out (for defecation) in the morning with the same people (childhood friends) for many years now*. *We share our stories*, *discuss our problems and plan our day during that time*.*" (42 years*, *female*, *FGD participant)*

#### Independent outdoor activity

It was reported that open defecation was considered as a personal activity by some respondents that made them feel independent as they could choose different venues, as they wanted on a regular basis. Open defecation was expressed as an activity that is done by active people.

"*… in addition*, *I can keep changing places whenever we want*. *This is one activity where I feel free to choose where to go*.*" (49 years*, *Male*, *FGD participant)*

#### Habit

Open defecation was perceived to be deeply influenced by the prevailing societal practice since historical times. Participants expressed open defecation as a regular habit for which they had never felt the need for alternatives.

"*I never imagined a day would come when people would use rooms for excreting…we enjoy the process in the serene environment beneath the open sky*.*" (70 years female*, *FGD participant)*

#### Convenient choice

One of the respondents expressed, not having a mental picture of oneself sitting in a latrine for defecation. As latrines are a new structure for him, the respondent expressed not knowing the process of latrine use. Thereby open defecation was taken as a more convenient choice.

*"We have never used latrine for years and we have grown up that way. I cannot think of ever sitting in a dark and stinky closed room to perform my daily activity (defecation)." (75 years, Male*, *FGD participant)*

#### Religious beliefs

Some participants argued the practice of open defecation continued from the times of gods and goddesses. Defecation nearby house (in latrines) would be against the religious and spiritual norms. The religious book not describing latrines was used to justify that open defecation was in line with the religious norms.

*"Oh my God! how can I defecate in my courtyard where I have been planting the religious Tulsi plant for years? It would be like slapping our religion. I am better off going nearby riverbanks." (60 years Male*, *IDI participant)*

#### Hygiene issues

Defecating in a latrine nearby their home was considered a source of disease nearby home. Open defecation was considered better hygiene as the dirt is left far away from home and the nuisance smell would not be found around their home.

### Motivations for open defecation by compulsion (issues regarding private latrines)

#### Absence of latrine at home

Female participants had more concerns regarding the lack of female voice to change of practice. The reason for open defecation was the lack of latrines at home. Given the choice, they would give up open defecation practice.

*"Our family is against building latrines at home, citing it to be against the tradition. We have no choice, no voice, but to go out (open defecation) before the darkness disappears in the morning." (19 years, Female*, *FGD participant)*

Despite feeling the need of a latrine for defecation, the respondents expressed that they defecated in the open because of the lack of resources to build a latrine. The resources ranged from financial to materials and land ownership. While the local government and non-government programs gave some financial and material incentives, the respondents reported a lack of additional funds, including maintenance plans for the latrine and the area around the house for latrine construction.

*“We feel ashamed that many of our neighbours have latrines and use them, but we don’t due to lack of funds. Open defecation costs us nothing.” (40 years male*, *IDI participant)*

*“Our family is too large that we don’t have even sleeping rooms. Where could we find the space to build a latrine?”(40 years male*, *FGD participant)*

#### Alternate use of Latrine

Building a latrine in (perceived) inappropriate places around the house (for e.g. in front of the kitchen or nearby the household prayer places) were more reasons for continuing open defecation despite having a latrine at home. As rats infested the existing store, the participant stored grains and other household goods in the newly constructed concrete latrine.

*"Haha….we live in a house with leaky roofs despite the rains and storms. It is ridiculous to use the well-built concrete rooms to excrete. It’s better to use it as a storeroom.” (60 years male*, *IDI participant)*

#### Hygiene and maintenance issues

Some of the respondents continued open defecation despite having a latrine at their house due to nuisance smell from the latrine. While some expressed concerns over cleaning up after using, some expressed concerns over the maintenance of the latrine.

*"We did not have any idea before construction of the latrine, what nuisance the smell would be. . . .it feels like we excrete in our beds. So, none of us uses it." (35 years male*, *FGD participant)*

*"If we use latrine daily, then the collection tank would fill up early.” (60 years*, *male, FGD participant)*

#### Household norms for latrine use

While respondents were comfortable going for open defecation, they expressed latrine in their house to be used by their special guests, male members, elderly or only during the nighttime.

*"We continue to go out for defecation (despite the newly built latrine) because we want the latrine to remain new and clean for guests, as we cannot afford to build another one if this gets old sooner." (30 years, male*, *IDI participant)*

*"The latrine at home is only used at night or by someone who is ill and cannot go out (for defecation). Regular use of latrine will pollute it sooner." (35 years*, *female, IDI participant)*

#### Cultural norms for latrine sharing

Female respondents expressed that it was against the norms for females of the house to share latrines with the male in the house; therefore, they were compelled to go out for defecation despite having a latrine at home.

### Motivations for open defecation by compulsion (issues regarding public latrines)

#### Issues with queuing

Regarding the use of public latrines as an alternative to open defecation, some respondents reported public latrines to be time-consuming. Some also reported that it would hamper their work to wait for long hours in the queue just to defecate.

*"I am happy to go out to defecate than waste my time waiting in the line*. *I would not waste my time just to excrete the waste." (45 years, Male, FGD participant)*

#### Privacy Issues for females

All female respondents raised concerns over privacy in using the public latrines. Female members are not comfortable being seen by male members while waiting in queues in front of the public latrines.

*"Oh, how could we stand in front of all males in the society to defecate?" (30 years female*, *IDI participant)*

#### Hygiene Issues

Using public latrine also meant that it would be dirty. It was expressed as a very unpleasant feeling to see somebody else’s dirt before using a latrine. Participants also expressed it as very uncomfortable to sit in a dark room with an unpleasant smell.

*"I do not like cleaning up after someone else’s dirt. Public latrines are too dirty." (52 years male*, *IDI participant)*

#### Cultural norms for latrine sharing

Latrine sharing between the males and the females was not common within the family. The females also highlighted this as a barrier for public latrine use. As there was a need for male members and female members to share the same public latrines, the female respondents who continued open defecation citing that the public latrines were inconvenient for females.

## Discussion

Defecation practices are associated with cultural and traditional beliefs [34], therefore exploring the reasons for the ongoing open defecation practices are essential. Understanding the motivations for open defecation may help future sanitation campaigns to help promote latrines at a community level. This study identified the motivators for open defecation by choice or by compulsion. People chose open defecation as a mode of socialization, an activity that gave a sense of autonomy, a habit, and a convenient choice. Religious beliefs and hygiene issues were also cited reasons for choosing open defecation. People were compelled to go for open defecation due to not having a latrine at home or having a latrine structure that was used for alternate purposes (e.g. storing grains). Other reasons compelling open defecation are the fear that latrine would get dirty quicker, the latrine is for used only for guests, or on certain occasions or time of the day; and the existing cultural norms of not sharing latrines between male and female members. People with access to public latrines were still compelled to go out for defecation due to the issues related to long queuing, hygiene issues as the public latrines are usually dirty and the cultural norms of latrine sharing restriction between male and female members of the society. Additionally, females also reported that they did not want to be seen by the male members while queuing in public latrines, which compelled them to go out in the open spaces for defecation. The research highlights a prominent gender component related to the practice of open defecation. The propriety still dictates that women and girls are still not considered of their opinion or needs for sanitary practices. Women and girls are compelled to go for open defecation either due to the absence of a latrine at home or they are not allowed to use the latrine at home as it to be used by the male members of the family. Women and girls also fear harassment or have privacy issues of being seen by the men from the community both during public latrine use and on the way to open defecation.

Concurring with the findings of our study, findings from India show that people who choose open defecation do so because they find open defecation to be more convenient, enjoyable [25] and healthy due to the long walks [35]. Furthermore, the same study from India reports that open defecation provides them with an opportunity for healthy habits of walking and socializing [25]. Open defecation is a good excuse for new brides to go out of the house and socialize with women in Uttar Pradesh, India [5]. It is likely for women and socially vulnerable men [36] to go for defecation in groups which helps them socialize and provides a sense of security from violence [5]. While our study reported a preference to men for using toilets at home while women were compelled to go outside, a study in India reports vice-versa phenomena where men prefer to go out for defecation. Some women consider open defecation as a medium for socialization, while men associate going out for defecation with their masculinity and they prefer that the women, children and the sick to use the toilet at home [5,35]. This clearly calls for researchers and policymakers to consider that open defecation has more personal and cultural aspects to it, which needs serious considerations during sanitation campaigns.

Personal and family/societal beliefs are reported to drive open defecation practices [34]. A report in 2017 presents similar factors as barriers for the use of latrine in Nepal [37]. The participants chose to defecate as far away from their house as possible in this study citing hygiene, religious and spiritual reasons. Some people were highly driven by religious beliefs for their open defecation behavior. However, religion was not mentioned as a reason for motivating open defecation in a similar setting in India [5]. A study from India also suggests people with social norms and beliefs rooted in purity and pollution prefer to defecate far away from home instead of having a latrine nearby their house [38]. A study from rural Odisha, India which has similar socio-cultural context with rural Nepal reports that, the women perceive open defecation as a way of disposing excreta away from home in order to minimize the chances of creating disease breeding places nearby home [27]. This finding is similar to this study where people chose open defecation as a choice to keep the house free from nuisance smell and diseases. In contrast to our study, participants in a Zambia report that using latrine is more hygienic as open defecation provides more opportunities for animals to come in contact with human feces providing an opportunity for disease transmission [16]. The findings from Zambia differ as the Zambian study was conducted in a Tenia endemic area where latrine use was encouraged to prevent Tenia infestation. This shows the need for identifying ways to disseminate health education regarding feco-oral transmission of diseases in rural Nepal to discourage open defecation. Behavior change in people is highly motivated by health concerns as reported for hand washing behaviors in a study in Nepal [39].

Open defecation is practiced as it is perceived more convenient compared to latrine use. Going to any convenient open space far away from home/community is perceived to provide people with a sense of independence, privacy and an opportunity to socialize. This finding is similar to the qualitative study on open defecation in India, where respondents report choosing open defecation places based on the availability of water source for cleaning and bathing after defecation, different areas in different seasons and women choosing far off areas to avoid passing by of a male pedestrian [27]. For women, privacy becomes the most important factor in choosing an open area for defecation.

Not having latrines due to lack of resources as reported in this study concurs with another report from 2017 from Nepal [37]. This is a gap that may be filled sooner with the help of the ongoing campaigns to support communities to build latrines in Nepal. It is not surprising that people with lesser resources would be less willing to build a latrine in their houses [27]. Using the latrine structure for storing grains has been discussed in a report from India as well. The same report mentions that people feel unsafe using latrines as there is a possibility of encountering snakes or spiders inside the latrine [5]. Therefore, campaigns must consider the personal and community motivators for open defecation to design interventions in order to promote the use of these constructed latrines.

Similar to our findings, the studies from rural Ethiopia and Zambia reported participants choosing to defecate in open areas and backyard instead of latrine due to the bad smell coming from the toilet, and also perceive defecating in a latrine as very strange and scary [16,40]. In a previous research in the study area [26], people with dirty latrine refrained from using latrines, which correlates with the current findings that bad smell and hygiene concerns regarding latrines motivate people for open defecation. A report from India strongly argued that interventions to identify and change the sanitary preference of the community along with the construction of latrines may be required to stop the ongoing open defecation [25].

The cultural norms restricting males and females to use the same toilet compel females to look for an alternative defecation places in open areas as reported in this study. This finding is further supported by a study in Nepal with reports of not allowing women to use the toilets used by men, as some communities perceive menstruation as a dirty event. Thereby women are not allowed to use the same toilet as men [37]. While the perceived untouchability during menstruation is being addressed by new laws in Nepal, further campaigns to address this issue in a culturally sensitive manner may be required. The cultural and social norms compelling male and female to use different defecation places was also reported from India [27].

As found in this study, public latrines are considered more inconvenient due to long queues and considered dirty. Women were concerned regarding safety, privacy and unhygienic sanitation in the public facilities. A similar finding was reported from women in Mozambique [41]. Women were not comfortable being seen by male members of the community queuing in public latrines. Open defecation is preferred choice over community/public latrine for women which is also reported from India [42]. Queuing in public latrine has been reported as an opportunity for male members to harass women in the community during the day. The report further elaborates that public latrines are closed at night, thereby open defecation was the only convenient choice [42]. Meanwhile, women in India reported harassment during open defecation including violence. However, the same study has no mention of harassment on women during queuing at the public toilets [5]. This reflects that different practices of defecation bring newer opportunities for harassment towards women and girls. Constructing separate public toilets for men and women may be a feasible option in this regards. However further research may be required to able be to recommend this.

Latrine coverage has increased in Nepal owing to the Government’s commitment to making an 'open defecation free' Nepal. National campaigns along with non-government partners provide support for building latrines in Nepal [21]. Despite this, some households are not able to build the latrine due to the lack of additional funds and spaces around the house. As suggested by the previous report, the government needs to promote self-initiation for latrines construction for improved utilization [26]. Therefore, there is a need for the local government to identify pragmatic interventions to motivate people to self-initiate latrine construction along with behavioral change interventions to address personal and social factors leading to open defecation at the community level.

### Strengths and limitations

This study provides reasons for open defecation, which was not adequately discussed in the existing quantitative reports. We conducted this study in the rural community where a previous research has identified factors influencing latrine utilization. Hence, this research further adds to the previous research. This study attempts to identify factors that may help ongoing campaigns to improve latrine construction and promote its use. The findings are useful for the policymakers working to address the ongoing open defecation in Nepal. More IDIs with local authorities, policymakers could have brought in additional perspectives for triangulation of the data. However, to address this we have used the maximum variation sampling to include respondents with different characteristics.

## Conclusions

Open defecation is chosen willingly or by compulsion by people in the communities. Understanding the personal preferences, perceptions, religious beliefs, family/cultural norms, societal practices, and availability of resources may help design better interventions for alleviating the practice of open defecation. There is a need to give careful considerations for understanding the motivations among females for open defecation in order to address open defecation and promote latrine use in a more gender equitable manner.

## Supporting information

S1 Table Data file. (PDF)

## Abbreviations

BPKIHS: B. P. Koirala Institute of Health Sciences
FGD: Focus group discussions
IDI: In-depth interview
LMIC: Low and middle-income countries
NDHS: Nepal Demographic Health Survey
ODF: Open defecation free
SDG: Sustainable development goals
