# Supplementary material for: What motivates open defecation? A qualitative study from a rural setting in Nepal
Source: PLoS One. 2019 Jul 1;14(7):e0219246. doi: 10.1371/journal.pone.0219246 (PMC6602253; doi:10.1371/journal.pone.0219246)
Supplement: S1 Table — (PDF) [file pone.0219246.s001.pdf]

Motivations for open defecation	
**By Choice**	
Socialization	• *F1: I have been going out (for defecation) in the morning with the same people (childhood friends) for many years now. We share our stories, discuss our problems and plan our day during that time. We discuss the current political situations. Accordingly, we also make a plan for our whole day during that precious time.*<br>• *K2: We get the opportunity to meet our friends while going in the morning for defecation. We generate different ideas to make our country prosperous.*<br>• *K5: I have never gone anywhere alone. I feel awkward and imagine how people would have been using that small dark room for defecating. Rather, I feel relaxed to excrete my waste in the open environment while talking with our friends at the same time.*<br>• *K10: Morning time is very important. We have to go to our work in the day time and can't meet our friends during the day. While meeting them during the morning, we can discuss the current socio-political problems of the country and generate ideas to mitigate those problems. This way, we have been saving our time.*<br>• *K13: Otherwise, we could not have met our relatives if we were absent during the morning defecation. It's like a morning update program for us.*

	• *F5: We feel interesting to talk about mischevious acts, about different relations between boyfriends and girlfriends in our morning sessions. How could we imagine to leave that interesting show?*<br><br>• *F13: We defecate which means making ourselves (our body) light. Meanwhile, we get the chance to share our emotions and personal issues which also make us feel light. So it's a double bonanza to make our body and mind light at the same time. It makes our day, as the saying 'Morning shows the day'*
Independent outdoor activity	• *F6: … in addition, I can keep changing places whenever we want. This is one activity where I feel free to choose where to go.*<br><br>• *F3: We feel bored to defecate daily at the same place. The momentum is great when you have places to change from time to time. Its really interesting.*<br><br>• *K17: You can't even imagine how it feels to defecate in the serene open environment.*<br><br>• *K16: Really saying, I can't adjust myself in that small dark congested room to defecate. You should feel good while defecating. So sorry, but I can't.*<br><br>• *K15: We usually go to defecation in the early morning. And it's an awesome feeling to defecate looking at stars in the sky.*<br><br>• *K19: I am an introvert person who loves to spend time alone in an open environment. I go early in the morning and generate ideas and create plans.*

Habit	• *F2: I never imagined a day would come when people would use rooms for excreting…we enjoy the process in the serene environment beneath the open sky.*<br><br>• *K20: I started defecating in the open place since birth. I have never imagined myself being defecating in the toilet.*<br><br>• *K13: In my view, Defecation means going to the riverside early in the morning, that's all.*<br><br>• *K14: I am quite lazy. Who would bother to carry the bucket full of water to the toilet…. (lazily…)*<br><br>• *K10: I had tried to defecate in my neighbour's toilet once. But I didn't urge to defecate there. After that, I have never used the toilet and dropped my idea of making the toilet in my house.*<br><br>• *K20: There is nothing to speak of. I just like to use the riverbanks to excrete. It's my habit and I don't prefer to change it.*<br><br>• *F7: I have a habit of chewing tobacco while defecating, otherwise, I don't have the urge to defecate. I usually spit the tobacco after the urge. If I use the toilet, I have to spit the tobacco in the toilet itself and it would be dirty soon. So, if I go to the open places, I can spit it anywhere anytime. I can't tolerate even a little nuisance smell.*<br><br>• *F9: I just love it…..(haha)…..for no reason.*

	K1: We are just two in my house. It's easy for us two to go to riverbank early in the morning, rather than building a new structure for defecating. Further, we are old enough and could die at any time.
Convenient choice	• *F7: We have never used latrine for years and we have grown up that way. I cannot think of ever sitting in a dark and stinky closed room to perform my daily activity (defecation).*<br>• *F7: You (…to the interviewer…) should try defecating in the open place for once. Then you would know the level of enjoyment. I am sure you would be preferring open defecation rather than using the toilet.*<br>• *K14: For using the toilet, you have to manage water, take it to the toilet, pour plenty of water in the pan. Who would bother to go through this long process? Rather a simple process, sit along the riverside, enjoy the defecation process, use the river water and return….haha…*<br>• *F8: The toilet pans are scary. One of my friend's son fell down from those sitting pads in the pan. He broke his leg. (….being furious….) I don't want my son to break his leg.*<br>• *K20: For defecating in the toilet, we need water. I don't want to touch the remains of my excreta at the anus while cleaning it with water. Rather we get big grasses near the river banks. We can use them to wipe the excreta at the anus. Later on, we can use water from the river to wash properly.*<br>• *K7: It smells terribly bad even when we pass flatus in*

	our living room. Imagine how it would smell if we defecate in a small room near our living room? I am not comfortable with this thing. Don't feel bad, but this is the truth and fact.
Religious beliefs	• *K6: Oh my God! How can I defecate in my courtyard where I have been planting the religious Tulsi plant for years? It would be like slapping our religion. I am better off going nearby riverbanks. (60 years Male, IDI participant)*<br><br>• *K20: Going to the toilet may be a good habit. But it can never be superior in front of our religion and religious believes.*<br><br>• *K6: Have u ever heard that our gods or goddesses used to go toilet for defecation? Have any religious books mentioned about the toilets? (…pause…….then continues….) Our existence is possible with god's grace. We are following what they enlightened us. And similar is the case with toilet use also. If gods are not using toilets, then who we are to go against them?*<br><br>• *F7: I have a joint family. My father worships our God and chants Mantras daily in the morning. I could hear them when I would be defecating. It's a great sin to come around the holy sound while defecating. It's a dire disrespect to our religion and our religious beliefs.*<br><br>• *F9: We are the son of priests. We have to wear the sacred thread (Janai) over our trunk throughout life. And you are well acquainted yourself that I should not teach you what is right or what is wrong…(worried face…). So*

	better not to ask such silly things.<br><br>• *F9: I had once gone to the neighbor's toilet for defecation. I used the toilet for 3 days. The day after that I fell very sick. I started having bad recurrent dreams. I fear even now when I remember that incidence. I went to the faith healers, they treated me for hours and concluded that my disease was due to the use of the toilet. After that, I left going to that toilet. Surprisingly I gradually recovered. Since then, I have never gone to the toilet and don't feel good to go there. It is not a good place for us to go.*<br><br>• *K20: Our ancestors used the river banks or the jungles for defecation. All our family lineage followed them. Now discontinuing this familial trait will be a huge disrespect for our ancestors. I don't want to break it.*<br><br>• *K6: We have to be clean and tidy all the time. Then only we can go to the temples. If we use the toilet, a huge amount of water is needed. You all know that there is a scarcity of water in our village. So, rather than being untidy, better to go to the river banks and use sufficient water.*
Hygiene issues	• *K14: I have heard that we get infected by improper sanitation through our toilet. We don't have enough water to pour in the toilet. (…….Thinking deeply…..nodding head)………So I am sure I and my family members cannot maintain the decorum of the toilet. Rather, its good to use open space which is far away from our house and is also very near to the river*

	bank. It’s easy for us there because in one way we are far from bacteria and there is less chance for them to invade our house, and on the other hand, no need to flush the stool in open space. And we can even use river water in case if needed.<br><br>• *F7: The nuisance smell produced by stool is terrible. And if it is present near to our house, how could we survive in that environment?*<br><br>• *K17: Open defecation has many benefits rather than the toilets. If we defecate our excreta in open places then after some times….(…humming…..) maybe after months…..our excreta would turn into fertilizer. So it will be good for plants to grow there. There’s a double bonanza on it. But people, who say themselves educated, don’t know this. It hard to make people understand this concept, but it’s the truth.*<br><br>• *F3: Children would go to the toilet and come back without flushing. Who would bother to go every time and pour a bucket of water? Rather not make a toilet, and go in open places.*<br><br>• *F9: I was actually planning to make a toilet in my house. Just a few days before I planned to make, I went to my maternal uncle’s house in another village. I lived there for 1 week. They had a toilet in their house. And to my surprise, it would smell so terribly bad that I could not eat food properly. After that, I dropped my idea of making a toilet in my house. Now I am going to the river bank which is far away from my house. There is no such problem of nuisance smell and I am happy with it.*

	• *F11: One of my friends made a toilet in his house. They started using it. Just 2 weeks after the use of the toilet, his children suffered from diarrhea and had to go to the hospital. They were hospitalized. But, till now, I and my family have been using an place for defecation and it is quite far from my residence area. Our family never suffered from any such hygienic diseases. Thus it's better to use an open place rather than making toilets in the house.*<br><br>• *F4: The main thing for being sick or the cause of diseases is improper sanitation and not washing hands with soap after defecation. I go to an open space far away from my house, dispose of my excreta properly by covering it with mud. Then I wash my hands with soap and water. Till now, I haven't suffered from any diseases yet. So it's not about making toilets. The important thing is proper washing of hands with soap and water.*
**By Compulsion**<br>**(Issues regarding Private Latrines)**	
Absence of latrine at home	• *F15: Our family is against building latrines at home, citing it to be against the tradition. We have no choice, no voice, but to go out (open defecation) before the darkness disappears in the morning.*<br>• *K5: "We feel ashamed that many of our neighbours have latrines and use them, but we don't due to lack of funds. Open defecation costs us nothing."*<br><br>• *F8: "Our family is too large that we don't have even sleeping rooms. Where could we find the space to build a latrine?"*

	• *F10: My house is just connected to the main road. There is no extra space to build a new structure around. I love to make a toilet, but I am constrained to use open space for defecation.*<br><br>• *F12: I told my parents about the importance of latrine use. I urged them to build a latrine in our house too. But it all went in vain. My parents say that they have been practicing open defecation for ages, yet they haven't suffered from any diseases. They don't have neither money nor any space around the house to build the latrine.*<br><br>• *F8: Sometimes, our desires also don't work. Our family has a desire to make a personal latrine for our home use. The local administration has assured to help us financially if we wanted to make the latrine in the house. But we don't have enough space in front of or beside my house to make it. Neither there is any free space surrounding my house where a latrine can be made. So I am compelled to use open space for defecation.*<br><br>• *F6: The administration provided us the ring for making a latrine, but we are unable to collect money even to complete the total structure of the latrine. I have started saving money daily to make a latrine, but I doubt how many years would it take to collect the total amount with this meager money collection.*
Alternate use of latrine	• *F11: Haha….we live in a house with leaky roofs despite the rains and storms. It is ridiculous to use the well-built concrete rooms to excrete. It's better to use it as a store room."*

	• *F11: When we built this house, we were only two-person living here. At that time, we also made a latrine beside my house. But now, my brother's family also resides with us. We are 6 now. It's extremely difficult for us to adjust 6 people in 2 rooms. And now we have too many materials to store as well. So to make our living easy, we put some wooden logs above the pan, covered it with a plain sheet of rubber and started keeping grains and other materials. It has been a bit easy for us these days. Otherwise, it would be very difficult for us. Also, the smell from the toilet was so annoying that we could not resist it. It's better now.*<br><br>• *K7: We used to live in a Kachcha house. The roofs started leaking since the previous year's summer. During rains, there was no option than to wet inside the room. We had no money either to make new building nor to repair the roofs. We have our latrine made of the concrete roof. Thus we closed our latrine by pouring mud inside the pan, closing the hole, making the pan fit with the surface of floor. Then we started living there. Now we are doing well. We are indeed the perfect example of survival of the fittest.*
Hygiene and maintenance issues	• *F11: We did not have any idea before construction of the latrine, what nuisance the smell would be. ...it feels like we excrete in our beds. So, none of us uses it.*<br><br>• *F14: You all know, there is a scarcity of water in this whole village. We seldom have proper drinking water. We have to bring water from far away river, and drink it after boiling. How could we use our toilet without the proper daily supply of water? We are worried about the hygiene and proper maintenance of the toilet. Thus, we prefer going to the open spaces, where nobody could see us.*

	• *K7: If we use latrine daily, then the collection tank would fill up early”*<br><br>• *F14: Haha….daily use of Latrine will make our latrine look old early. We don’t have enough money to repair it or build it again. So, we usually prefer going out in the early morning. There are other people as well who go with us in the morning. But at night time, when we have to use the latrine on the emergency, then we usually go to our toilet.*<br><br>• *F14: Our family members used the toilet most often. But, there are many neighbours who don’t have a toilet in their house. So they used to come to our house for defecation. And they didn’t clean properly and pour plenty of water after defecating. In this way, it started becoming dirty sooner. So we decided not to use ourselves also so that other people also don’t come these days. Now you can see, our toilet is clean. We occasionally use it when in need, when we cannot go to outer places.*<br><br>• *K7: I have a toilet in my house. But I rarely use it. My family also uses it rarely. The reason is that a few of my friends don’t have toilets in their house. They all march towards the riverbank early in the morning. When they start going out for defecation, it is still dark. At that time, they usually fear to go alone. So we have to accompany them during that journey. Otherwise, they would be angry with us. We have to care about our friendship too.*
Household norms for latrine	• *K12: We continue to go out for defecation (despite the*

use	*newly built latrine), because we want the latrine to remain new and clean for guests, as we cannot afford to build another one if this gets old sooner.*<br>• *K3: The latrine at home is only used at night or by someone who is ill and cannot go out (for defecation), if needed. Regular use of latrine will pollute it sooner.*<br><br>• *F14: If we start using the latrine daily, then children from the neighbouring houses would also start coming here. They don't clean the latrine properly, it smells awfully bad. So we also stopped ourselves from using the latrines. We only use it if it's a must need.*<br><br>• *K18: We go to work in the factory during the day time. There is no one in the house during that time. I will share a story. One day, I had gone to the factory for my work. My husband also goes with me at work. When we returned home in the evening, our toilet was filled with the pile of stool over the pan and flies were hovering around. We cleaned it that day. But to our surprise, it was repeated daily. Then we closed the toilet with a big lock. Since then we also haven't used it.*<br><br>• *F11: The toilet is attached to our house. We don't have enough water in the house to pour it in the toilet. So if we don't pour enough water, it smells dreadful. So we don't use it. Occasionally when there is a dire need, we use it.*
Cultural norms for latrine sharing	• *K4: Who would listen to us? We are always a minority at home. The bearer knows how the shoe pains. Our father and mother-in-law say that we should not use the same*

	toilet where they have been defecating. Otherwise, they would be sick soon. They are very superstitious. When we say this to our husband, they also take the side of their parents. Nobody listens to us. All our words go into vain. So we have stopped talking about it. Rather we go to some open spaces early in the morning.<br>• *F14: It's really difficult for us during menses. We are not allowed to touch anyone during menses. So, we have to reside separately. And using the same toilet is a far cry for us.*<br><br>• *K3: When we have menses, other family members think that we are untouchable. We have heavy bleeding at that time. So, they think that it will impure their home and don't allow us to use the latrine. So we are compelled to go out to open spaces for defecation or for urination.*
**By Compulsion**<br>**(Issues regarding Public Latrines)**	
Issues with queuing	• *K8: Oh, how could we stand in front of all males in the society to defecate in front of them?*<br>• *F4: …I am happy to go out to defecate than waste my time waiting in the line. I would not waste my time just to excrete the waste.*<br>• *F12: People start accumulating since the early morning for defecating in the public toilet. We have to work from the start of the morning to earn our livelihood. We have to prepare food for our children early in the morning since they go to school. Then we have to prepare ourselves for our work. Thus, we prefer going to open spaces rather*

	than waiting in a long queue.<br><br>• *F15: During the queue, people start talking about non-sense things. Females start talking about the personal issues of different family members and males start talking and scolding the politician for all the misdeeds. I hate these discussions in the early morning. I am a person of a different kinds. I enjoy being alone, and it works even in the case of defecating in the morning.*<br><br>• *F15: I feel shy to meet all those people, mostly elderly people of the village on the queue. I have no strength to face them all.*
Privacy issues for females	• *K18: There is only one public toilet in the village and it is common for both males and females. We feel very uncomfortable to face ourself among them in the early morning.*<br><br>• *F15: The males are busy talking rough things in the queue. They utter the vulgar jokes with their male counterparts. How could we hear them? It's very uneasy for us.*<br><br>• *F13: I have been suffering from Piles for many years. It takes a long time for me to finish the defecation. I feel shy to stand in front of those females and consume so much of time. I feel really bad. Thus I prefer going to the bushes where I can spend my time freely without any fear and shy.*

Hygiene issues	• *K10: I do not like cleaning up after someone else's dirt. Public latrines are too dirty.*<br>• *K19: Not all people are similar and not all people have similar habits. The public toilet is used by so many people. Some of the people make the toilet so dirty and smelly that the smell is very awful. If you go after them for defecating in that public toilet, you cannot tolerate the nuisance smell coming there. Your urge to defecate gets lost. So it's better to go out openly in free spaces.*<br>• *F12: There is no person to clean after the public toilet. The administration made the public toilet but hasn't hired a person taking care of. The toilet gets filthy if not cleaned each day. I lose my urge to defecate on that situation. It's the reason I prefer going to open spaces or the river banks or the bushes.*<br>• *F3: There is no proper place for people to wait in the queue. We have to stand just in front of the toilet and wait for the person to come out. Waiting there, just in front of the toilet, the smell is offensively bad.*
Cultural norms for latrine sharing	• *F15: There are many superstitious people in our village who don't want to see female involvement in public forums. They are not happy with females coming in between the male talks. So, we feel shy to go to the public toilets most often.*<br>• *F15: The men tease us when they see us. We don't feel comfortable to go to public toilets.*<br>• *F9: I lost my leg 5 years ago in an accident in the factory. The public toilet is not disabled friendly.*

	So we prefer going to open spaces and sit comfortably and defecate. I would be much happy to use the toilet if it becomes easy for us to use it.<br>•
